# Comprehensive analysis of neuronal guidance cue expression regulation during monocyte-to-macrophage differentiation reveals post-transcriptional regulation of semaphorin7A by the RNA-binding protein quaking

**DOI:** 10.1177/1753425920966645

**Published:** 2020-11-26

**Authors:** Huayu Zhang, Jurriën Prins, Dianne Vreeken, Barend W Florijn, Ruben G de Bruin, Oscar RJ van Hengel, Mieke F van Essen, Jacques MGJ Duijs, Hilde Van Esch, Eric P van der Veer, Anton Jan van Zonneveld, Janine M van Gils

**Affiliations:** 1 Einthoven Laboratory for Vascular and Regenerative Medicine, Department of Internal Medicine, Leiden University Medical Centre, The Netherlands; 2 Department of Human Genetics, University Hospitals Leuven, Belgium

**Keywords:** Monocyte, macrophage, semaphorin, quaking, microRNA

## Abstract

In response to inflammatory cytokines and chemokines, monocytes differentiate into macrophages. Comprehensive analysis of gene expression regulation of neuronal guidance cue (NGC) ligands and receptors in the monocyte-to-macrophage differentiation process is not available yet. We performed transcriptome profiling in both human primary PBMCs/PBMC-derived macrophages and THP-1 cells/THP-1-macrophages using microarray or RNA sequencing methods. Pathway analysis showed that the axonal guidance pathway is significantly regulated upon monocyte differentiation. We confirmed NGC ligands and receptors which were consistently regulated, including SEMA4D, SEMA7A, NRP1, NRP2, PLXNA1 and PLXNA3. The involvement of RNA-binding protein quaking (QKI) in the regulation of NGC expression was investigated using monocytes and macrophages from a QKI haplo-insufficient patient and her healthy sibling. This revealed a positive correlation of SEMA7A expression with QKI expression. *In silico* analysis of 3′UTRs of NGCs proposed the competitive binding of QKI to proximal microRNA targeting sites as the mechanism of QKI-dependent regulation of SEMA7A. RNA immunoprecipitation confirmed an interaction of QKI with the 3′UTR of SEMA7A. Loss of SEMA7A resulted in monocyte differentiation towards a more anti-inflammatory macrophage. Taken together, the axonal guidance pathway is regulated during monocyte-to-macrophage differentiation, and the regulation is in line with the necessary functional adaption for the specialised role of macrophages.

## Introduction

Monocytes, with their ability to phagocytose, present Ags, and produce cytokines, play important roles in immune surveillance. In response to inflammatory cytokines and chemokines, they are able to extravasate through endothelium and differentiate into macrophages, which are more specialised in phagocytosis and play an significant role in innate immunity.^1^ Besides their important roles in various physiological processes, macrophages are responsible for the pathophysiology of various inflammatory diseases, including atherosclerosis, Crohn’s disease and various neurodegenerative diseases.^2^ Upon monocyte-to-macrophage differentiation, the cells change their functionalities in multifaceted ways, which can partly be reflected by altered gene expressions in biological pathways. A comprehensive understanding of biological pathway regulations during monocyte-to-macrophage differentiation gives insights into their functions in health and disease.

Neuronal guidance cues (NGCs) are ligands in the axon guidance pathway that trigger downstream signalling by binding to their receptors. They consist of four classes –semaphorin (SEMA), netrin (NTN), ephrin (EFN) and slit (SLIT) – with their receptors being plexin/neuropilin (PLXN/NRP), UNC5/DCC/NEO1, EPH and ROBO, respectively. Functionally, these guidance cues affect adhesion, migration and activation of cells. In recent years, we have learned that NGCs play an important role in immunology and are also specifically shown to be involved in monocyte–macrophage biology. For example, SEMA7A has been shown to stimulate production of pro-inflammatory cytokines in monocytes, and potentially promote differentiation of monocytes to macrophages.^3^ NTN1 and SEMA3A have been found to inhibit migration of monocyte-like cell lines and adhesion of leucocytes to capillary endothelium.^4^ In ApoE knockout mice fed a high-fat diet, NTN1 inhibited emigration of macrophages from atherosclerotic lesions.^5^ To interpret the involvement of NGCs better in monocyte-to-macrophage differentiation, we sought out to give an overall image of the expression of guidance cues and their regulations during monocyte-to-macrophage differentiation using multiple commonly used experimental models.

Quaking (QKI), an RNA binding protein (RBP), has been identified as a central regulator of gene expression changes in the monocyte-to-macrophage differentiation process.^6^ One of the mechanisms through which QKI performs its regulatory functions is that QKI binds to quaking response element (QRE) motif in mRNA stabilising mRNA transcript.^7,8^ The QRE motif consists of the core sequence ‘ACUAA’, often followed by a half site ‘UAAY’.^7^ Despite the central role of QKI in monocyte-to-macrophage differentiation, involvement of QKI in the regulation of NGCs is yet to be studied.

In the current study, we found significant regulation of genes in the axonal guidance pathway during monocyte-to-macrophage differentiation by pathway analysis. Consistent regulation of gene expression was identified among multiple experimental models. In addition, we explored the involvement of QKI in the regulation of NGC ligands and receptors.^6^ Using material from a QKI haplo-deficient patient, we demonstrated the possibility that regulation of SEMA7A was dependent on the competitive binding of QKI to a microRNA (miRNA) targeting cluster in 3′UTR of SEMA7A.

## Methods

### Culture of human monocytes and THP-1 cells

THP-1 cells were obtained from ATCC (TIB202), and were cultured in RPMI1640 medium with 10% FBS supplemented with 0.01 μg/ml l-glutamine, penicillin and streptomycin and 0.05 mM 2-mercaptoethanol. For differentiation of THP-1 cells to THP-1 macrophages, 100 nM phorbol 12-myristate 13-acetate (PMA; Sigma–Aldrich) was added to the medium. After 3 d, the PMA-containing medium was replaced by normal growth medium, and the cells were cultured for another 5 d.

Primary human monocytes were isolated from whole blood donated by the QKI haplo-deficiency patient and her sibling. PBMCs were isolated by gradient centrifugation in Ficoll-Paque (Sigma–Aldrich). Monocyte fractions were then labelled with anti-CD14 Ab conjugated to magnetic beads (Miltenyi) and separated on a MACS magnetic column, according to the manufacturer’s instructions. The purity of the isolated monocytes was around 95% (Supplemental Figure S1). Isolated monocytes were either subjected directly to RNA isolation procedure or differentiated to pro-inflammatory macrophages in RPMI 1640 medium with 10% FBS supplemented with 5 ng/ml recombinant human granulocyte-macrophage colony-stimulating factor (GM-CSF; Biosource). The medium was refreshed every 2 d until d 7.

Ethical issues regarding utilisation of patient-derived samples have been described previously.^6^ Briefly, informed consent was obtained prior to the experiments, and the proposals were approved by relevant ethics committees at the Maastricht University Medical Centre, The Netherlands, and Leuven University Hospital, Belgium.

### Omics data access

Transcriptome (GSE9820,^9^ last downloaded on 15 January 2015) and miRNA (GSE52986, last downloaded on 1 October 2018^10^) profiling data of human monocytes were obtained from the Gene Expression Omnibus (GEO) repository using the ‘GEOquery’ package from R bioconductor, according to the developer’s manual.^11^ QKI eCLIP data were downloaded from the ENCODE website (ENCSR366YOG, last downloaded on 23 September 2018).^12^ A summary of in-house and online data used in this article is provided in Supplemental Table S1.

### Gene expression microarrays

A detailed method has been described before by de Bruin et al.^6^ THP-1 cells and THP-1 macrophages were lysed in Trizol (Thermo Fisher Scientific). RNA was isolated using the RNeasy Mini Kit (Qiagen) according to the manufacturer’s instructions. Complementary DNA synthesis was done with the WT Expression Kit (Affymetrics). Fragmentation and terminal labelling were done using the WT Terminal Labeling Kit (Affymetrics). The library was then hybridised to HJAY Chips (Affymetrix 540091). Chips were scanned using the Affymetrix Gene ChIP Scanner 3000 7G (Affymetrix). Probe intensity data were analysed using the standard Affymetrix pipeline according to the manufacturer’s instructions. Data were deposited in GEO under accession number GSE74887.

### RNA sequencing

RNA sequencing analysis (RNA-seq) was performed, as previously described.^6^ Briefly, ribosomal RNA was removed using Ribo-Zero rRNA Removal Kit (Illumina). Sequence libraries for mRNA were generated using TruSeq RNA Library Prep Kit (Illumina). The cDNA fragments, ∼300 bp in size, were isolated and purified using 2% E-gel SizeSelect gels (Invitrogen). The libraries were then pair-end sequenced on an Illumina HiSEQ platform to a depth of 41–70 million reads per sample. The sequencing results were mapped using Tophat2 software to GRCh37/UCSC-hg19 genome assembly with the gene model provided by ‘hg19 UCSC Known Genes’.^13^ Guidance cues with non-zero counts were included for subsequent analyses.

### Pathway analysis

Pathway analysis was done using annotation of the Kyoto Encyclopedia of Genes and Genomes (KEGG)^14^ with the R package ‘gage’.^15^ Gene symbols were converted to Entrez ID using the database provided in the R package ‘org.Hs.eg.db’. Pathway ‘Axon guidance’ (KEGG accession: hsa04360) was tested under the null hypothesis that this pathway is not significantly regulated. A *P* value of < 0.05 was considered sufficient for rejecting the null hypothesis. The analysis was visualised using the R package ‘pathview’.

### Quantitative PCR

RNA isolation was done as described for the gene expression microarray method. Briefly, RNA isolation was done using the miRNeasy Mini Kit (Qiagen), while random hexamers served as primers for first strand cDNA synthesis. The PCR was run on a CFX384 Real-Time PCR Detection System, and SYBRgreen (Applied Biosystems) was used to detect amplification of the PCR product. A list of primer sequences can be found in Table 1. We analysed four housekeeping genes in the THP-1 monocytes and macrophages. Three out of four were unchanged by differentiation (Supplemental Figure S2a). From this, *GAPDH* was chosen for the analysis of primary monocytes differentiated to macrophages. Comparing different donors showed that *GAPDH* mRNA did not change upon differentiation (Supplemental Figure S2b). Therefore, the presented change in mRNA expression was calculated by the comparative copies per *GAPDH*.

**Table 1.** Primers used.

Symbol	Forward primer (5′→3′)	Reverse primer (5′→3′)
ACTB	TGCGTGACATTAAGGAGAAG	TGAAGGTAGTTTCGTGGATG
ADORA2B	GGGCTTCTGCACTGACTTCT	CCCGTGACCAAACTTTTATACCTG
EPHB3	GTCATCGCTATCGTCTGCCT	AAACTCCCGAACAGCCTCATT
GAPDH	TTCCAGGAGCGAGATCCCT	CACCCATGACGAACATGGG
IL10	GCGCTGTCATCGATTTCTTCC	GTAGATGCCTTTCTCTTGGAGCTTA
NRP1	GGCGCTTTTCGCAACGATAAA	TCGCATTTTTCACTTGGGTGAT
NRP2	AAGTTCACCTCCGACTACGC	CATGCACGTTTCAAGCCCTC
PLXNA1	TGTGCAGTACACGTCCTGTG	GCTCGTCTGCTCGCTCAC
PLXNA3	CGGACATGTTCAGTCTCGTGTA	CGCTGACGAAGCCGTAGAT
PLXND1	CTGTGCATGTGGAGTGATGG	GTTCCTTCCTCGGATGGTCAG
QKI5	GACTGGCATTTCAATCCAC	GATGGACACGCATATCGTG
RHOA	GATTGGCGCTTTTGGGTACAT	AGCAGCTCTCGTAGCCATTTC
RPS18	CCTGCGGCTTAATTTGACTC	AGACAAATCGCTCCACCAAC
SEMA3C	TGTGAAAGGATCTTCCCAGCC	GATGGTGGGAAAGGCTGAAGT
SEMA4A	CTCCCCACATCTACGCAGTC	AGAGAGAAGGCACAAACCGC
SEMA4B	GAGCGGCCATTCCTCAGATTC	CACCCACGTACAGGGTCCT
SEMA4D	GACAGCGATGGCATTTGCAC	CCTCCCGGGCACCTATGTA
SEMA4F	TCCAATCTCTGAGGCTGACTC	GGGATAAAGCGAAGATGGTGTC
SEMA7A	ATCTTCGCCGTCTGGAAAGG	TAGTTCTCGCAGTCCGTGC
SEMA7A 3′UTR	GGCATCGATGACCCAAGACT	TGGGAGCATCGCTGCATTTA
TNFA	CCTCTCTCTAATCAGCCCTCTG	GAGGACCTGGGAGTAGATGAG

### Mapping of ‘ACUAA’ 5mer and miRNA target sites in 3′UTR of transcripts

Genome coordinates of 3′UTRs of NGC transcripts were retrieved from Ensembl annotation ‘EnsDb.Hsapiens.v75’^16^ using the R package ‘GenomicFeatures’.^17^ Sequences of 3’UTRs were obtained using the R package ‘ensembldb’.^18^ Motif ‘ACUAA’ was matched against 3’ UTR sequence using the R package ‘Biostrings’.^19^ Counts of the 5mer were stored and related to the regulation of NGCs. Genome coordinates of QRE motifs were also stored for subsequent analysis. To examine the possibility that QKI could protect 3′UTRs of NGCs from targeting miRNA, we collected genomic coordinates of miRNA target sites from ‘miRbase.org’^20^ and of QRE 5mers from the previous mapping. The miRNA target sites were filtered by the miRNA expression levels given from the monocyte miRNA microarray (GSE52986^10^; Supplemental Table S1 data set 4; expression level in top 50%) and by miRNA targeting strength score (miRsvr score < –1). QRE mappings were cross-validated by experimentally confirmed interaction of QKI and target mRNA using eCLIP-seq method (ENCSR366YOG^12^) by overlaying genomic range of narrow peak signals to QRE mappings. Next, we scanned for proximity of validated QRE and filtered miRNA targeting sites using the R package ‘GenomicRanges’.^17^ We considered a physical distance of less than 20 nucleotides as proximity. Final results were visualised with the R package ‘Gviz’.^21^

### RNA immunoprecipitation

HEK293T cells were transfected with a human QKI5 and human SEMA7A 3′UTR-reporter plasmid (SwitchGear Genomics) through the use of polyethylenimine (3 µg/1 µg DNA; Polysciences, Inc.). Twenty-four h after transfection, RNA-immunoprecipitation was performed using Millipore’s validated RIPAb+QKI-5 kit according to the manufacturer’s instructions.

### Immunoblot analysis

Suspended cells were collected, spun down, washed with cold PBS and lysed in 100 µl Novex Tris-Glycine SDS sample buffer (Thermo Fisher Scientific; LC2676). Adhered cells were washed with cold PBS, lysed in another 100 µl sample buffer, collected and combined with lysates from suspended cells. After sonication, samples were centrifuged at 20,000 *g* for 10 min at 4°C. Equal amounts of protein samples, measured with Nanodrop (Thermo Fisher Scientific), were denatured using DTT and heating at 95°C for 10 min followed by size separation on a 10% Mini-PROTEAN gel (Bio-Rad; 4561033). Proteins were transferred to PVDF membranes (Bio-Rad; 1074156) using the Trans-Blot Turbo system (Bio-Rad) after which membranes were blocked in TBST-5% milk. Membranes were incubated with human SEMA7A Ab (1:10,000, AF 2068; R&D systems) or GAPDH Ab (1:1000, 5174; Cell Signaling Technology) in blocking buffer overnight at 4°C. Thereafter, membranes were shortly washed and incubated with HRP-labelled secondary Abs (1:5000; Dako) for 1 h at room temperature. After thorough washing with TBST, protein bands were visualised using Western lightning ECL (PerkinElmer; NEL1030001EA) and the ChemiDoc Touch Imaging System (Bio-Rad).

### Statistical analysis

Comparison between two means was done with a two-tailed unpaired Student’s *t*-test. A *P* value of < 0.05 was considered significant.

## Results

### Axonal guidance pathway is significantly altered in monocyte-to-macrophage differentiation

In order to assess the relevance of the axon guidance pathway in monocyte-to-macrophage differentiation, we extracted transcriptome profiling data of human PBMCs and monocyte-derived macrophages (Supplemental Table S1 data set 1) and of THP-1 cells and THP-1 macrophages (Supplemental Table S1 data set 2). KEGG pathway analysis on the two data sets (Supplemental Figures S3 and S4) showed significant (*P* = 0.016 and 0.007, respectively) regulation of gene expression in the axon guidance pathway (KEGG: hsa04360) during monocyte-to-macrophage differentiation (Table 2). Since downstream signalling in the axon guidance pathway is initiated by binding of guidance cues and their receptors on the cell surface, we next focused on the regulation of NGC ligands and their receptors during monocyte-to-macrophage differentiation.

**Table 2.** Pathway analysis of the transcriptome change in experimental monocyte–macrophage differentiation.

Pathway	Accession	Biological process	*P* Value
Axon guidance	KEGG: hsa04360	Human monocyte–macrophage	0.016
Axon guidance	KEGG: hsa04360	THP1–THP1 macrophage	0.007

### Detectable expression of NGCs in monocytes and macrophages

**Figure 1.**
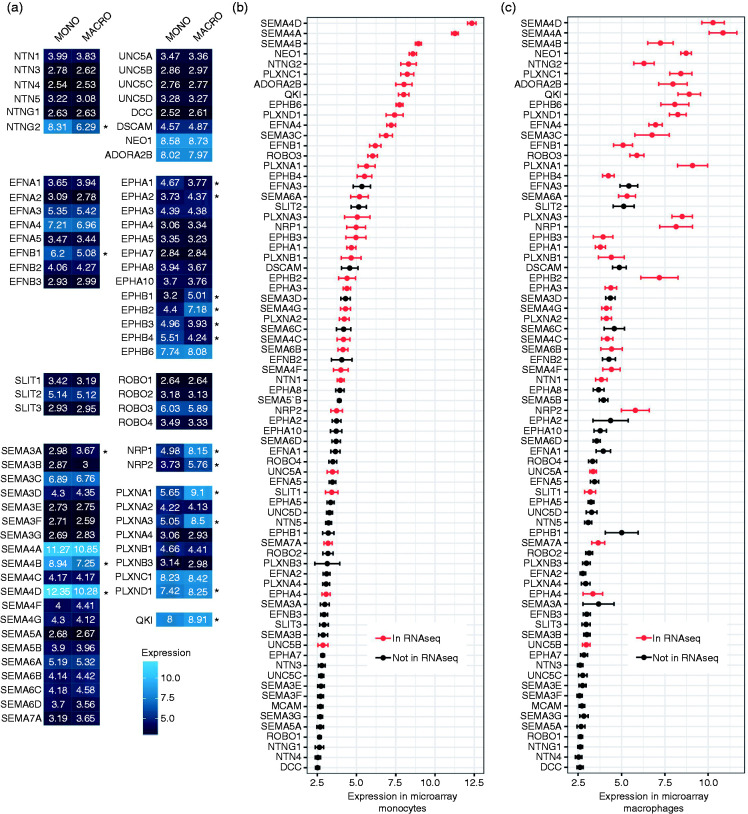
Expression of NGCs in human monocytes/macrophages. (a) Heat map of mRNA expression of NGCs in human monocytes/macrophages detected by microarray expressed in log2 scale (lighter blue indicates higher expression; *n* = 12; *P < 0.05). (b) and (c) Average expression levels of mRNA NGCs in human monocytes (b) or macrophages (c) detected by microarray ranked by expression in monocytes from high to low. RED: detectable in RNA-seq. Data are mean ± SEM; *n* = 12. NGC: neuronal guidance cue.

To gain insight in the genes with significant expression, we summarised the transcriptome data obtained from both microarray and RNA-seq methods. Significant expression of NGCs from different classes was detected in primary human monocytes and macrophages (Figure 1a and Supplemental Table S1 data set 1) and in THP-I cells and THP-I macrophages (Supplemental Figure S5 and Supplemental Table S1 data set 2). To examine the comparability between observations of the two methods, we plotted the relative probe signal intensity, representing expression of NGCs in descending order, and differentially labelled the ones that were detectable in RNA-seq data (Figure 1b and c). As we have *n* = 3 with the microarray method, the signals from the microarray methods give a good overview of the expression of NGCs and its variation. The RNA-seq experiment, as a method with better sensitivity and larger dynamic range, provides extra information on the likelihood of whether NGCs with borderline expression are actually expressed. Several NGCs, including SEMA4D, SEMA7A, NRP1, NRP2, PLXNA1, PLXNA3 and NTN1 that are known to be involved in monocyte–macrophage physiology, could be detected with both the microarray and RNA-seq. However, some NGCs such as EPHA4 and UNC5B were detected with RNA-seq but have a low expression in the microarray. This might be due to the intrinsic character of a higher dynamic range of RNA-seq. Overall, it can be concluded that numerous NGCs are expressed by monocytes and macrophages.

### Regulation of NGCs in monocyte-to-macrophage differentiation

**Figure 2.**
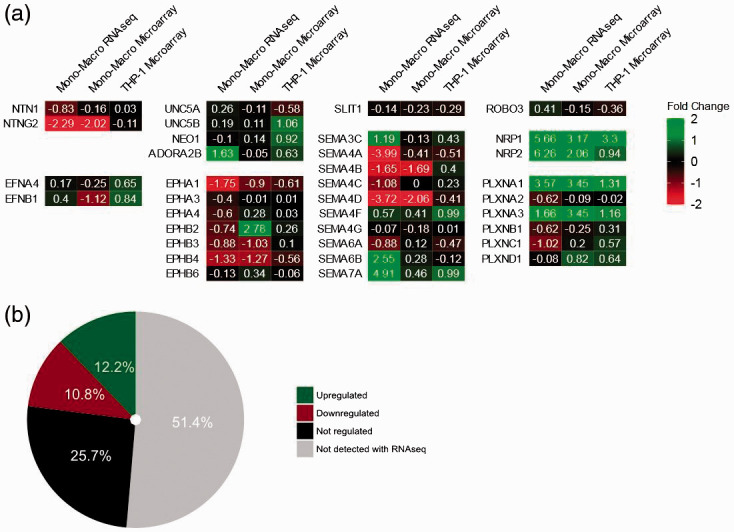
Change of NGC expression during monocyte-to-macrophage differentiation. (a) Heat map of fold change of NGC expression in log2 scale (green: up-regulation; red: down-regulation; mono–macro RNA-seq *n* = 1; mono–macro microarray *n* = 12; THP-1 microarray *n* = 3). (b) Summary of NGC expression regulation during monocyte/macrophage differentiation.

**Figure 3.**
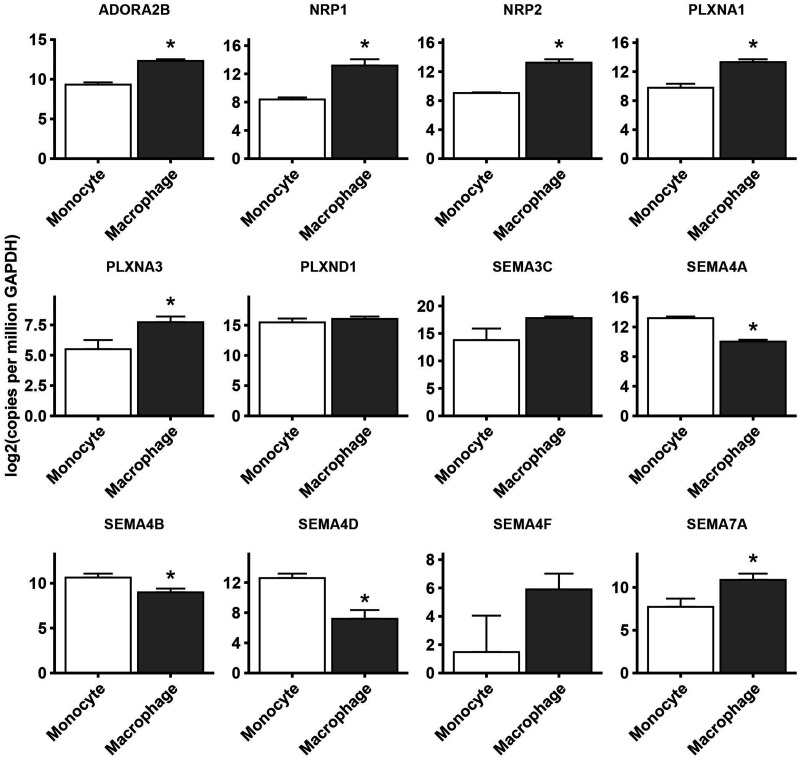
Confirmation of regulation of NGC expression. Quantitative PCR analysis of NGCs mRNA isolated from human monocytes or macrophages. Gene expression is expressed as copies per GAPDH in a log2 scale. Data mean ± SEM; *n* = 3; *P < 0.05.

Next, we assessed the changes in expression of NGCs in monocyte-to-macrophage differentiation *in vitro* in three datasets (mono–macro RNA-seq, mono–macro microarray and THP-1 microarray). Fold changes in NGC expression in macrophages relative to monocytes are depicted in a heat map and are shown in Figure 2a. Around half of all NGCs were detectable with RNA-seq (36/74; Figure 2b). Of the detected NGCs, 12.2% were up-regulated (9/74), while 10.8% were down-regulated (8/74) in at least two of the three data sets (Figure 2a and b). Regulation of NGC expression was confirmed by qPCR (Figure 3 and Supplemental Figure S6). The list of regulated genes is shown in Table 3, and a brief annotation of the known relevant functions is also provided. Overall, it can be concluded that the results obtained from different methods had good agreement with each other.

**Table 3.** Overview of regulated genes and their known relevant function.

Gene	Mono-mϕ RNA-seq	Mono-mϕ microarray	THP-1 microarray	Expression	Type	Annotation
NTNG2	Down	Down*	–	11	Netrin ligand	Function unknown
ADORA2B	Up	–	Up*	111	Netrin receptor	ADORA2B has a pro-inflammatory role through production of cAMP^22^
EPHA1	Down	Down*	Down*	11	Ephrin receptor	EPHA1 is a receptor for EFNA1, which promotes monocyte adhesion^23^
EPHB3	Down	Down*	–	1	Ephrin receptor	EPHB3 is a receptor for EFNB2, which promotes monocyte adhesion^24^
EPHB4	Down	Down*	Down*	1.	Ephrin receptor	EPHB4 is a receptor for EFNB2, which promotes monocyte adhesion^24^
SEMA3C	Up	–	Up*	11111	Semaphorin	SEMA3C enhances deformability of DCs, promoting migration of DCs^25^
SEMA4A	Down	Down	Down	111111	Semaphorin	SEMA4A promotes expression of VEGFA in monocytes^26^
SEMA4B	Down	Down*	–	1111	Semaphorin	Function unknown
SEMA4D	Down	Down*	Down*	11111.	Semaphorin	AntiSEMA4D treatment augments pro-inflammatory function of mϕ^27^ SEMA4D itself also promote differentiation of monocyte to alternatively activated mϕ^28^
SEMA4F	Up	Up*	Up*	.	Semaphorin	Function unknown
SEMA6A	Down	–	Down*	1.	Semaphorin	SEMA6A signals via PLXNA4 leading to repulsion of cell migration^29^
SEMA7A	Up	Up*	Up*	1	Semaphorin	SEMA7A works as a stimulator of monocytes, promoting the production of GM-CSF^3^
NRP1	Up	Up*	Up*	11	Neuropilin	NRP1 is a co-receptor of Class III semaphorins, which are repellents for monocyte adhesion and migration^4^ NRP1+ mϕ migrate towards SEMA3A expressed in hypoxic niches of tumours^30^
NRP2	Up	Up*	Up*	11	Neuropilin	NRP2 is a co-receptor of Class III semaphorins, which are repellents for monocyte adhesion and migration.^4^ NRP2 is required for phagocytosis of mϕ^31^
PLXNA1	Up	Up*	Up*	11	Plexin	PLXNA1 is a receptor of Class III semaphorins, which are repellents for monocyte adhesion and migration^4^ PLXNA1 facilitates migration of tumour-associated mϕ into hypoxic niche^30^
PLXNA3	Up	Up*	Up*	1.	Plexin	PLXNA3 is a receptor of Class III semaphorins, which are repellents for monocyte adhesion and migration^4^
PLXND1	–	Up*	Up*	11111	Plexin	SEMA3E-PLXND1 axis promotes mϕ infiltration in adipose tissue^32^

Annotation of functions of the NGCs that showed consistent changes in at least two of the three monocyte-to-macrophage differentiation data sets.

*P < 0.05).

mϕ: macrophages; DC: dendritic cells; NGC: neuronal guidance cue; Expression: expression level indicated with 111111 being highest and lowest.

### QKI contributes to the regulation of NGCs

**Figure 4.**
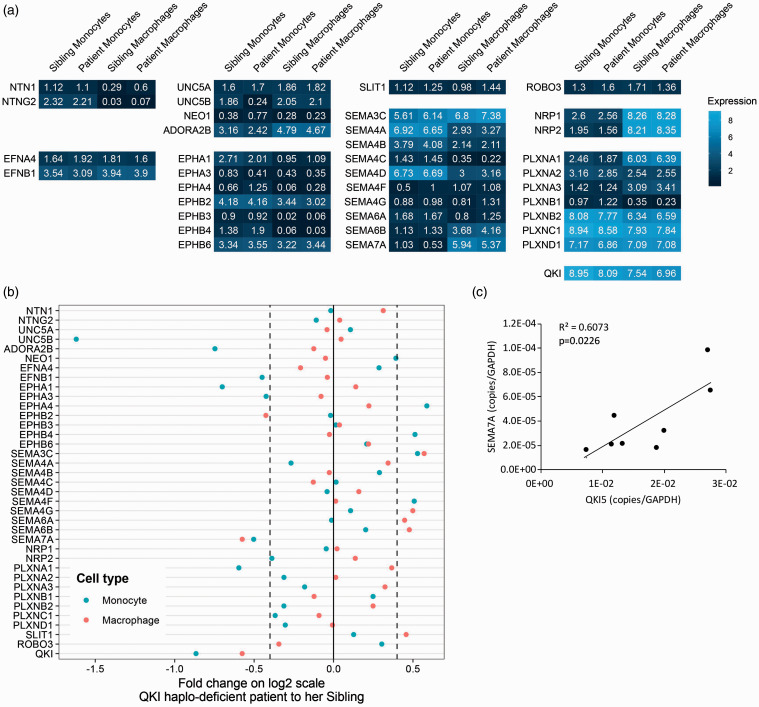
Regulation of NGC expression by QKI. (a) Heat map of expression of NGCs in monocytes and macrophages determined by RNA-seq from QKI patient and sibling in log2 scale (Lighter blue indicates higher expression; *n* = 1). (b) Fold change of NGC expression comparing QKI patient’s monocyte and macrophage to her sibling’s. (c) In macrophages treated with GapmeR antisense oligonucleotides, there is a significant positive correlation between QKI and SEMA7A expression measured by quantitative PCR analysis. Gene expression is expressed as copies per GAPDH. QKI: quaking.

Next, we investigated the role of the RNA-binding protein QKI in the regulation of NGC expression. Similar to NGCs, QKI was originally identified for its function in the central and peripheral nervous system, while further studies revealed that QKI was ubiquitously expressed.^8^ In addition, QKI is known as one of the key master regulators controlling gene expression change during monocyte-to-macrophage differentiation.^6^ QKI is up-regulated in pro-inflammatory macrophages, modulating the expression of various genes that have important functions in macrophage biology. To gain insight into whether QKI is implicated in regulating NGC expression in monocyte-to-macrophage differentiation, we exploited material from a patient with a haplo-deficiency of QKI (patient) and her unaffected sibling. The haplo-deficiency is caused by chromosomal translocation that occurred in the intron of the *QKI* gene. The transcription of a complete *QKI* gene is disrupted, leading to reduced *QKI* expression. Transcriptome profiling was done using the RNA-seq method in both freshly isolated PBMCs and monocyte-derived macrophages (Figure 4a and Supplemental Table S1 data set 3). Due to the limitation of numbers of samples by nature, we could only examine the change of NGC expression with an arbitrary threshold (± log_2_0.4). Fold changes of NGC expression comparing the patient and her sibling showed SEMA7A to be consistently regulated in monocytes and monocyte-derived macrophages (Figure 4b). Expression of SEMA7A was decreased in the patient’s monocytes and macrophages compared to the monocytes and macrophages from her sibling. To extend the results obtained with the QKI haplo-deficient patient, we reduced QKI expression in primary human monocytes harvested from healthy controls using GapmeR antisense oligonucleotides that either targeted QKI for degradation or are scrambled as a control.^6^ The GapmeR antisense oligonucleotides were present during the differentiation of the primary monocytes to macrophages. Although the GapmeR-mediated reduction in QKI expression varied a lot per donor, it nonetheless enabled us to validate a positive correlation between QKI and SEMA7A expression (Figure 4c).

### QKI binds to cluster of miRNA target sites in SEMA7A 3’UTR

**Figure 5.**
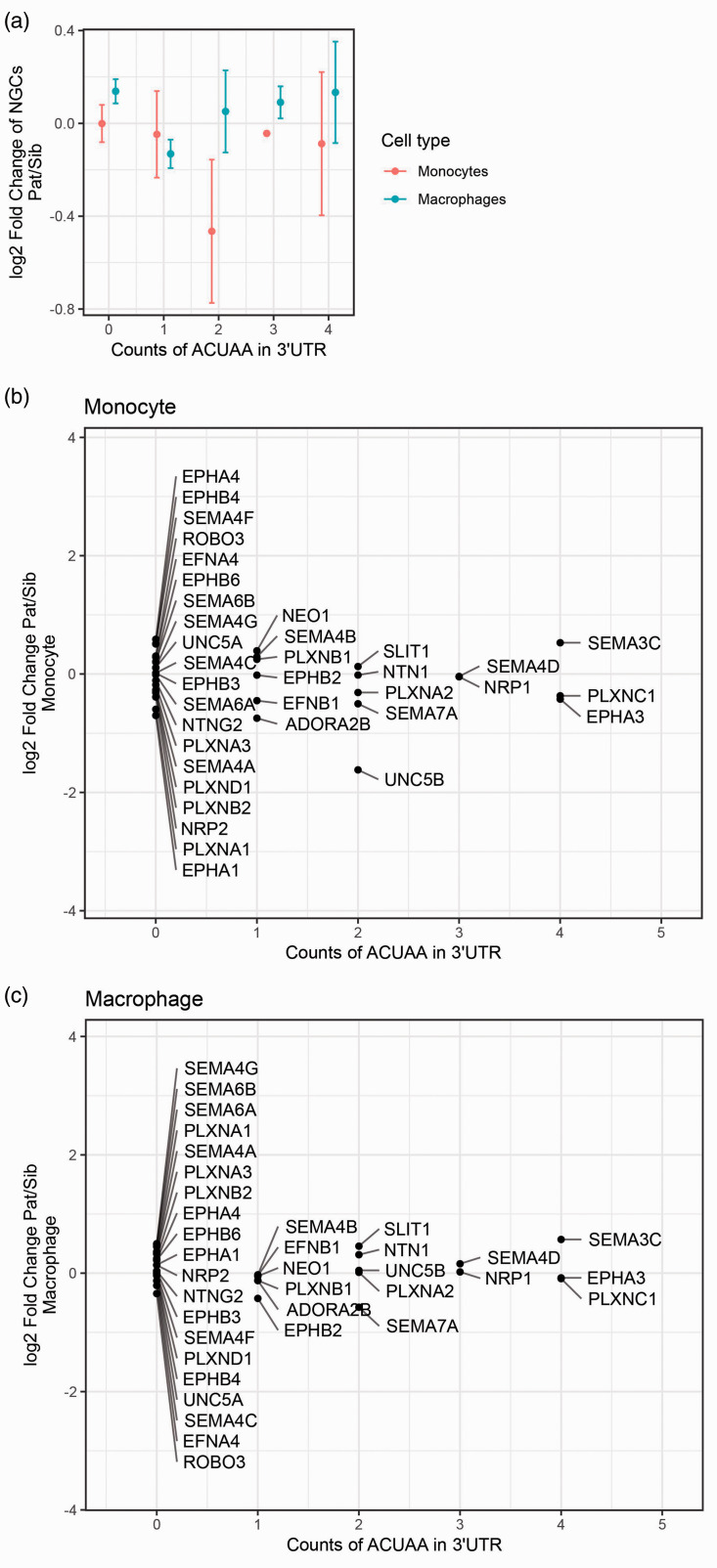
QKI response elements in NGC transcripts. (a)–(c) Scatter plots of QRE motif counts in the 3′UTRs of NGCs against log2 fold change of NGC expression in monocytes and macrophages. (a) Monocytes and macrophages combined. (b) and (c) Monocyte shown separately with individual NGCs. Existence of QKI response element (QRE/ACUAA motif) did not correspond with the fold change of NGC expression in the QKI haplo-deficient patients.

**Figure 6.**
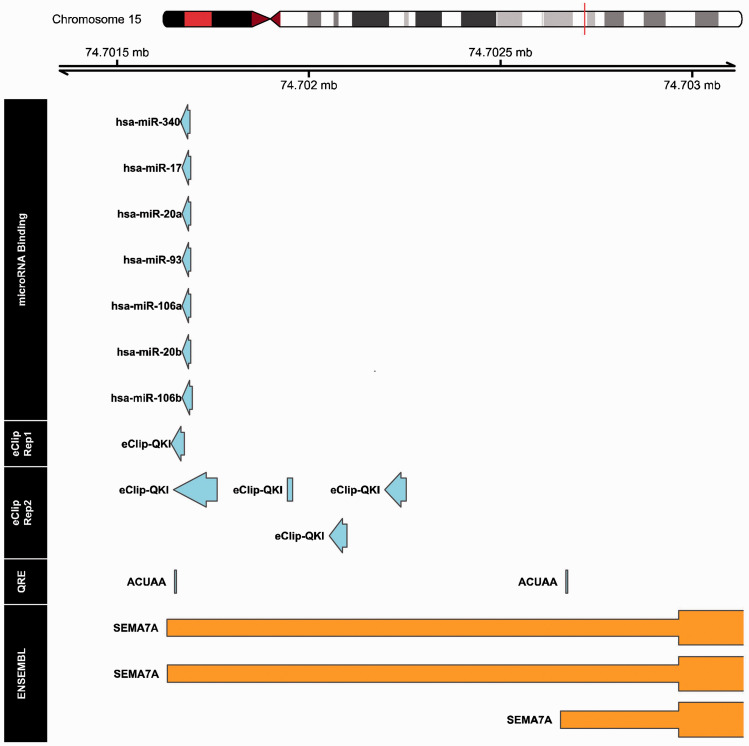
Visualisation of genomic annotations in 3′UTR region of *SEMA7A* gene. The region in 3′UTR of SEMA7A in the human genome (hg19 Chromosome 15 q24.1, bp 74,701,500–74,703,000) is annotated with aspects of miRNA binding sites, QKI targeting sites (experimental evidence based), QRE (*in silico* alignment of ‘ACUAA motif’) and SEMA7A exon. The microRNA binding information was extracted from ‘miRbase’ and was filtered by expression in monocytes/macrophages and miRNA interaction score. Interactions between RNA binding proteins (RBPs) to genomic DNA were obtained from technique duplicates of eCLIP-seq in the myelogenous K562 cells.

**Figure 7.**
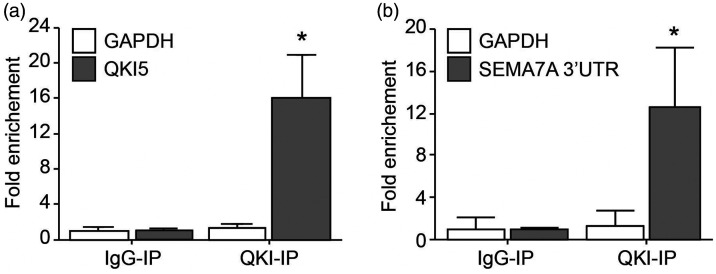
RNA-immunoprecipitation showing quaking protein binding to the 3′UTR of SEMA7A. (a) RNA-immunoprecipitation in Hek293T cells overexpression QKI5 using an IgG control or QKI5 Ab. QKI5 and GAPDH mRNA abundance in immune-precipitated fraction was determined by qPCR. Results are presented relative to IgG immunoprecipitation. Data are the mean ± SEM; *n* = 5; *P < 0.05. (b) RNA-immunoprecipitation in Hek293T cells overexpression QKI5 and 3′UTR of SEMA7A using an IgG control or QKI5 Ab. SEMA7A 3’UTR and GAPDH mRNA abundance in immune-precipitated fraction was determined by qPCR. Results are presented relative to IgG immunoprecipitation. Data are the mean ± SEM; *n* = 3; *P < 0.05.

One of the well-defined mechanisms by which QKI affects gene expression is to stabilise gene transcripts by binding to QRE motifs on their 3′UTRs.^7,8^ A QRE consists of a core sequence ‘ACUAA’ and a half site ‘UAAY’.^7^ We therefore explored the fold change of NGC expression between the patient and her sibling in relation to the number of ‘ACUAA’ core sequences in 3′UTRs of NGCs (Figure 5a and b). Surprisingly, the analysis revealed no clear correlations between the fold change of the NGCs and QRE counts, so that existence of the QRE sequence alone did not explain the effect of QKI to enhance expression of SEMA7A. We then explored the possibility of whether binding of QKI to 3′UTRs of mRNAs might mask the sites for miRNA interactions by scanning proximal pairs of miRNA targeting sites and QRE motifs in 3′UTRs of NGC genes. The scanning results revealed that only in the 3′UTR of the *SEMA7A* gene was such proximity between QREs and miRNA targeting sites present (Figure 6). The QRE at the very end of SEMA7A’s 3′UTR overlaps with QKI–DNA interactions sites from both duplicates from eCLIP-seq, increasing the confidence that this QRE is actively used. In the proximity of the QRE, a targeting site of a cluster of miRNAs exists. It is therefore probable that QKI and miRNAs antagonistically regulate expression of SEMA7A in a proposed broader concept of RBP–miRNA interaction. Moreover, the QRE was experimentally verified with CLIP-seq methods in the K562 myeloid cell line (Figure 6 and Supplemental Table S1 data set 5). This observation further supported our theory that QKI enhances SEMA7A transcript stability by competing with the binding of miRNAs in the 3′UTR of SEMA7A. We next sought to determine whether QKI can directly bind to 3′UTR of SEMA7A using RNA-immunoprecipitation experiment. For this, we used Hek293T cells in which both QKI and a construct containing the 3′UTR of SEMA7A were overexpressed. QKI is known to bind RNA of QKI itself, which we also observed (Figure 7a). Indeed, when analysing the SEMA7A 3′UTR, RNA of SEMA7A 3′UTR was highly enriched in the QKI-Ab precipitated RNA fraction (Figure 7b), indicating binding of QKI to the 3′UTR of SEMA7A.

### SEMA7A induced monocyte differentiation towards a pro-inflammatory macrophage

**Figure 8.**
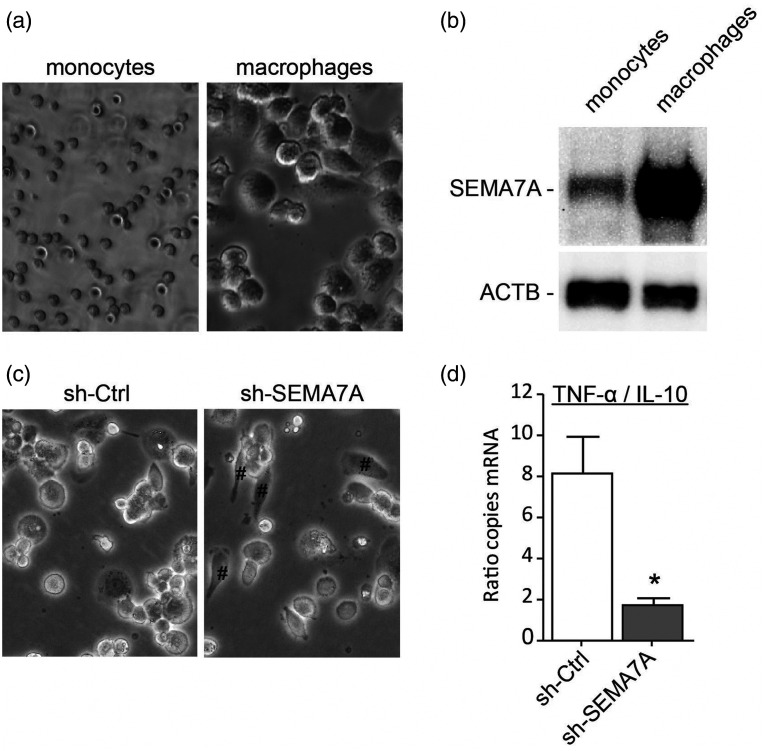
Role for SEMA7A in monocyte differentiation into pro-inflammatory macrophages. (a) Bright field microscope images from THP-I monocytes and macrophages. (b) Representative immunoblot analysis of SEMA7A or ACTB (loading control) in protein lysates of THP-I monocytes and macrophages. (c) Bright field microscope images from THP-I macrophages transduced with anti-SEMA7A shRNA (sh-SEMA7A) or scrambled shRNA (sh-Ctrl). Elongated phenotype indicated by #. (d) Ratio of TNF-α and IL-10 mRNA expression of sh-SEMA7A or sh-Ctrl THP-I macrophages. Data are the mean ± SEM; *n* = 4; *P < 0.05.

Before further exploring the functional importance of SEMA7A for macrophages, we validated the increase of SEMA7A on monocyte-to-macrophage differentiation seen at mRNA level at protein level (Figure 8a and b). To gain insight into the function of SEMA7A in monocyte-to-macrophage differentiation, SEMA7A expression in monocytes was silenced using lentiviral shRNA vectors targeting the SEMA7A mRNA and non-SEMA7A targeting control shRNA. These monocytes were differentiated into macrophages. Targeted reduction of SEMA7A in monocytes did not seem to affect the capacity of the monocytes to differentiate into macrophages, but did show more elongated anti-inflammatory macrophage morphology (Figure 8c). To confirm the hypothesis that loss of SEMA7A induced a more anti-inflammatory phenotype, we investigated expression levels of TNF-α (pro-inflammatory macrophage cytokine) and IL-10 (anti-inflammatory macrophage cytokine). Indeed, SEMA7A knockdown macrophages showed a ratio that was more anti-inflammatory compared to control macrophages (Figure 8d).

## Discussion

In this study, we identified the axon guidance pathway as a novel pathway regulated in the monocyte–macrophage differentiation process. We illustrated the regulation of NGCs and annotated the shift in functionality contributed by the regulation of axon guidance pathway based on the currently available knowledge from the literature. We also investigated the involvement of QKI in regulation of SEMA7A in a miRNA-dependent manner.

The different platforms analysed to detect NGC gene expression in human monocytes and macrophage showed numerous NGCs expressed. NTN1 was low but significant expressed. It was detectable with the RNA-seq method, and had a signal above background in the microarray. The relevance of NTN1 secreted from macrophages has been shown to inhibit their emigration in an autocrine or paracrine manner.^5^ NGCs such as EPHA4 and UNC5B were detected with RNA-seq, but had low expression in microarray. This could be due to the intrinsic character of RNA-seq that it has a larger dynamic range. We found many members of the semaphorin family expressed by monocytes and macrophages, both ligands and receptors. For example, SEMA4D (CD100), an immune semaphorin, was identified with both methods, and has been shown to mediate monocyte adhesion and differentiation.^28,33^ Expression of the SEMA7A–PLXNC1 ligand–receptor pair, which was recently shown to be involved in atherogenesis,^34^ was also detected.

During the differentiation of both human primary monocytes to macrophages and THP-1 cells to THP-1 macrophages, up-regulation of receptors for Class III semaphorins, namely NRP1, NRP2, PLXNA1 and PLXNA3, was observed. Previous studies have shown that activation of the SEMA3–PLXNA–NRP axis is reduced in monocyte–endothelium interactions, although SEMA3A–NRP1 binding could sustain directed migration of macrophages under hypoxic condition in tumour tissues.^4,30^ Up-regulation of these receptors could sensitise the response of macrophages to the guidance signals provided by Class III semaphorins. In addition, we observed down-regulation of membrane bound Class IV semaphorins, namely SEMA4A, SEMA4B and SEMA4D, in macrophages compared to monocytes. SEMA4D is known to promote differentiation of alternatively activated macrophages, while anti-SEMA4D treatment increases the population of classically activated macrophages in mammary carcinoma tissue, and induces the production of inflammatory cytokines.^27,28^ This is in line with our finding of SEMA4D down-regulation in macrophages, considering GM-CSF stimulation is a model of monocyte-to-classical macrophage differentiation. In contrast to SEMA4D, SEMA7A expression was increased in monocyte-to-macrophage differentiation. Our studies indicate that SEMA7A itself serves as a stimulator for monocytes to differentiate into a pro-inflammatory macrophage. These observations are in line with the findings of Holmes et al. that SEMA7A results in an increased production of inflammatory cytokines and GM-CSF, which could be a potential positive feedback mechanism for monocyte activation and differentiation.^3^ Indeed, neutralisation of SEMA7A using an Ab reduced the number of pro-inflammatory macrophages in a murine viral myocarditis model.^35^

QKI was shown to be a critical regulator of monocyte-to-macrophage differentiation.^6^ During the differentiation, expression of QKI increases, triggering a range of effects, including transcript stabilisation and alternative splicing. We investigated whether regulation of some NGCs could be mediated by QKI by comparing the NGC expression in monocytes and macrophages of the QKI haplo-deficient patient and her sibling. As described previously, SEMA7A was up-regulated in monocyte-to-macrophage differentiation. The comparison of the patient and her sibling revealed that SEMA7A was decreased in both monocytes and macrophages. QRE motifs were found in 3′UTR of the SEMA7A transcript, which are necessary for the transcript stabilisation effect of QKI. However, counts of QRE motifs alone could not explain the regulation of SEMA7A expression by QKI activity, indicating that extra factors are involved in the regulation of SEMA7A by QKI. It has been proposed that QKI could stabilise transcripts of miRNAs, thereby regulating gene expression.^36^ However, such a mechanism is unlikely to be applicable in our case, since SEMA7A would have been up-regulated in patients if any miRNA targeting SEMA7A was stabilised by QKI. Therefore, we explored the QRE motif region, and found a cluster of miRNA target sites in proximity of QRE. This observation pointed to the possibility that the competitive binding of QKI to miRNA targeting sites is the underlying mechanism for regulation of SEMA7A by QKI. Such a concept has been reported for other RBPs before^37,38^ but not yet for QKI. We now show that QKI binds to the 3′UTR of SEMA7A. Further studies are needed to confirm the competitive access of QKI and miRNA to 3′UTR of SEMA7A to be responsible for the observed changes in SEMA7A expression.

In summary, during monocyte-to-macrophage differentiation, the axonal guidance pathway was significantly regulated, with changes in expression of several NGC ligands and receptors. Regulation of NGCs in monocyte-to-macrophage differentiation fits the functional adaption that is necessary for the specialised role of macrophages – so far, a role for SEMA7A in regulating macrophage polarisation into a pro-inflammatory macrophage. In the future, the changes in gene expression can be further studied in the context of diseases where monocyte–macrophage biology plays a role.

## Supplemental Material

sj-pdf-1-ini-10.1177_1753425920966645 - Supplemental material for Comprehensive analysis of neuronal guidance cue expression regulation during monocyte-to-macrophage differentiation reveals post-transcriptional regulation of semaphorin7A by the RNA-binding protein quaking Click here for additional data file. Supplemental material, sj-pdf-1-ini-10.1177_1753425920966645 for Comprehensive analysis of neuronal guidance cue expression regulation during monocyte-to-macrophage differentiation reveals post-transcriptional regulation of semaphorin7A by the RNA-binding protein quaking by Huayu Zhang, Jurriën Prins, Dianne Vreeken, Barend W Florijn, Ruben G de Bruin, Oscar RJ van Hengel, Mieke F van Essen, Jacques MGJ Duijs, Hilde Van Esch, Eric P van der Veer, Anton Jan van Zonneveld and Janine M van Gils in Innate Immunity

sj-pdf-2-ini-10.1177_1753425920966645 - Supplemental material for Comprehensive analysis of neuronal guidance cue expression regulation during monocyte-to-macrophage differentiation reveals post-transcriptional regulation of semaphorin7A by the RNA-binding protein quaking Click here for additional data file. Supplemental material, sj-pdf-2-ini-10.1177_1753425920966645 for Comprehensive analysis of neuronal guidance cue expression regulation during monocyte-to-macrophage differentiation reveals post-transcriptional regulation of semaphorin7A by the RNA-binding protein quaking by Huayu Zhang, Jurriën Prins, Dianne Vreeken, Barend W Florijn, Ruben G de Bruin, Oscar RJ van Hengel, Mieke F van Essen, Jacques MGJ Duijs, Hilde Van Esch, Eric P van der Veer, Anton Jan van Zonneveld and Janine M van Gils in Innate Immunity
