# Supplementary material for: Comprehensive analysis of neuronal guidance cue expression regulation during monocyte-to-macrophage differentiation reveals post-transcriptional regulation of semaphorin7A by the RNA-binding protein quaking
Source: Innate Immun. 2020 Nov 26;27(2):118–32. doi: 10.1177/1753425920966645 (PMC7882812; doi:10.1177/1753425920966645)
Supplement: sj-pdf-1-ini-10.1177_1753425920966645 - Supplemental material for Comprehensive analysis of neuronal guidance cue expression regulation during monocyte-to-macrophage differentiation reveals post-transcriptional regulation of semaphorin7A by the RNA-binding protein quaking [file sj-pdf-1-ini-10.1177_1753425920966645.pdf]

Supplementary Figure legends

Supplementary Figure 1. Assessing purity of monocyte isolation. A sample of peripheral blood mononuclear cells (top), of which the CD14-positive selected cells (middle) where isolated and the non-selected cells (bottom) were incubated with anti-human CD14 (APC, 1:200, Clone MφP9, cat no. 345787, BD), measured by flow cytometry LSR-II (BD Biosciences, Vianen, The Netherlands), and analyzed using FlowJo Software version 10 (Tree Star, Ashland, OR, USA).

Supplementary Figure 2. Assessing multiple housekeeping genes. Quantitative PCR analysis of different housekeeping mRNA isolated from THP1-monocytes or -macrophages (A) or primary monocytes or macrophages (B). Gene expression is expressed Ct values. (A) each color is different housekeeping gene measured in 3 independent THP1 monocytes and 3 independent THP1 macrophages samples. (B) each color represents a different donor of primary monocytes or macrophages, $n = 7$.

Supplementary Figure 3. Visualization of the axon guidance pathway and the gene expression changes during human monocyte-to-macrophage differentiation. The gene expression in monocytes and macrophages was obtained from GEO database (Accession: GSE9820, Supplementary Table 1 Dataset 1). Data from 12 individuals were pooled for each cell type.

Supplementary Figure 4 Visualization of the axon guidance pathway and the gene expression change during THP-1 cell to THP-1 macrophage differentiation. The changes in gene expression in THP-1 cells to THP-1 macrophages were profiled in on the "Affymetrix GeneChip Human HJAY Array" (Accession: GSE74887, Supplementary Table 1 Dataset 2). Data from 3 technical replicates were pooled for each cell type.

Supplementary Figure 5. Expression of NGCs in human monocyte/macrophage cell line. Heatmap of expression of NGCs in THP1 cells/THP1 macrophages in a log2 scale. Light blue: higher expression; dark blue: lower expression; $n = 3$; * $P < 0.05$.

Supplementary Figure 6. Confirmation of regulation of NGC expression. Quantitative PCR analysis of NGCs mRNA isolated from THP1-monocytes or -macrophages. Gene expression is expressed as copies per GAPDH in a log2 scale. Graphs show the mean ± S.E.M. $n = 3$ * $P < 0.05$.

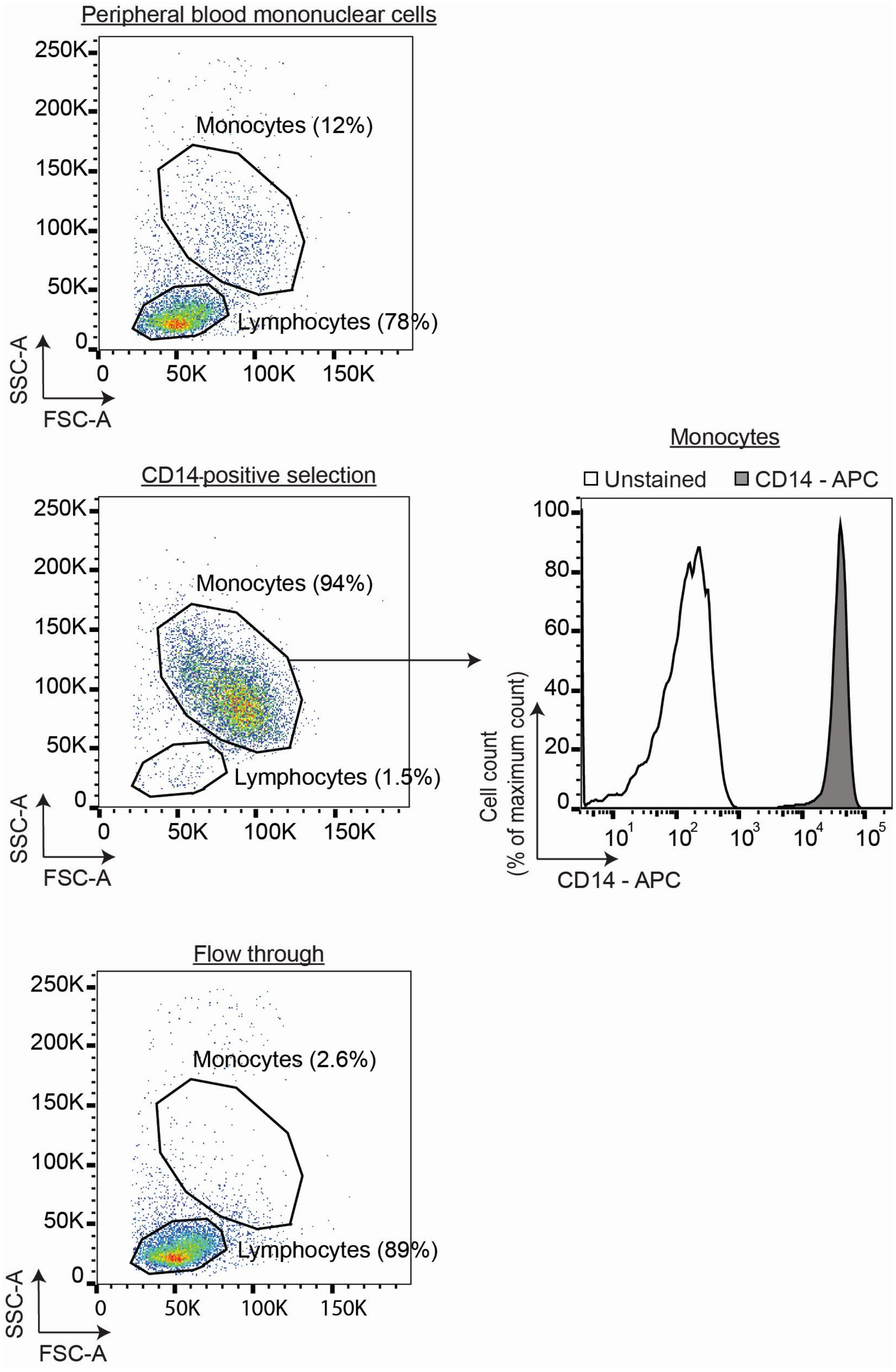

Peripheral blood mononuclear cells
Monocytes (12%)
Lymphocytes (78%)
250K
200K
150K
100K
50K
0
0
50K
100K
150K
SSC-A
FSC-A
CD14-positive selection
Monocytes (94%)
Lymphocytes (1.5%)
SSC-A
FSC-A
Monocytes
Unstained
CD14 - APC
100
80
60
40
20
0
10^1
10^2
10^3
10^4
10^5
Cell count
(% of maximum count)
CD14 - APC
Flow through
Monocytes (2.6%)
Lymphocytes (89%)
SSC-A
FSC-A

Figure 1

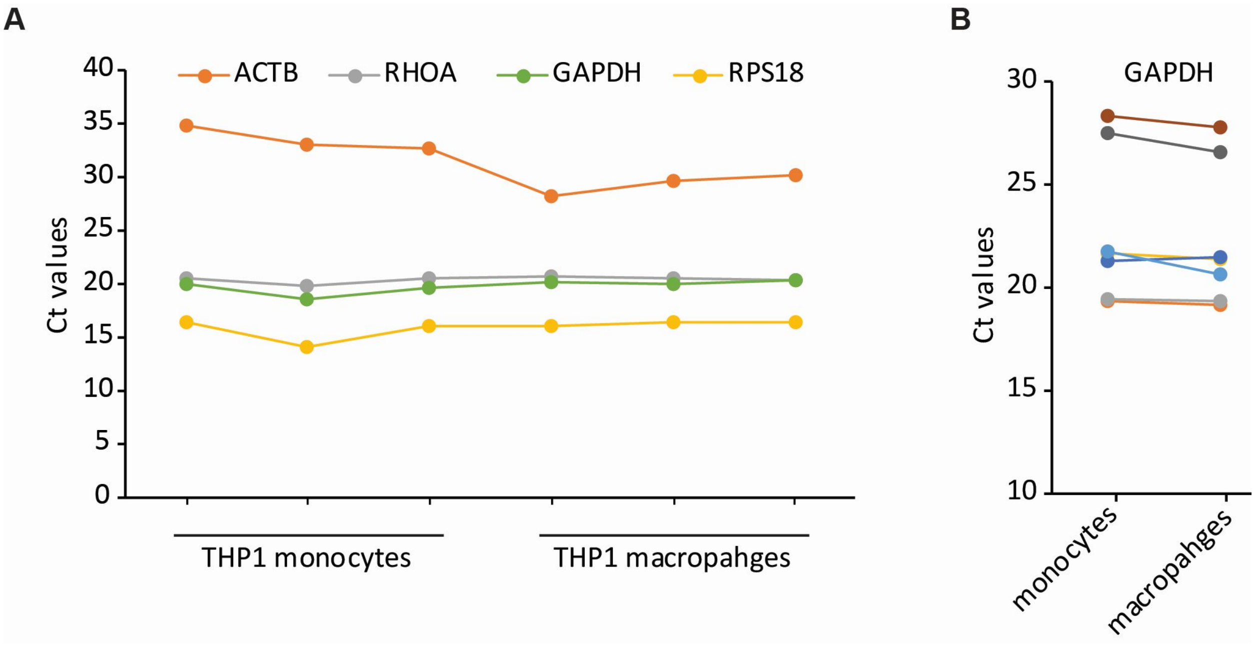

Figure 2

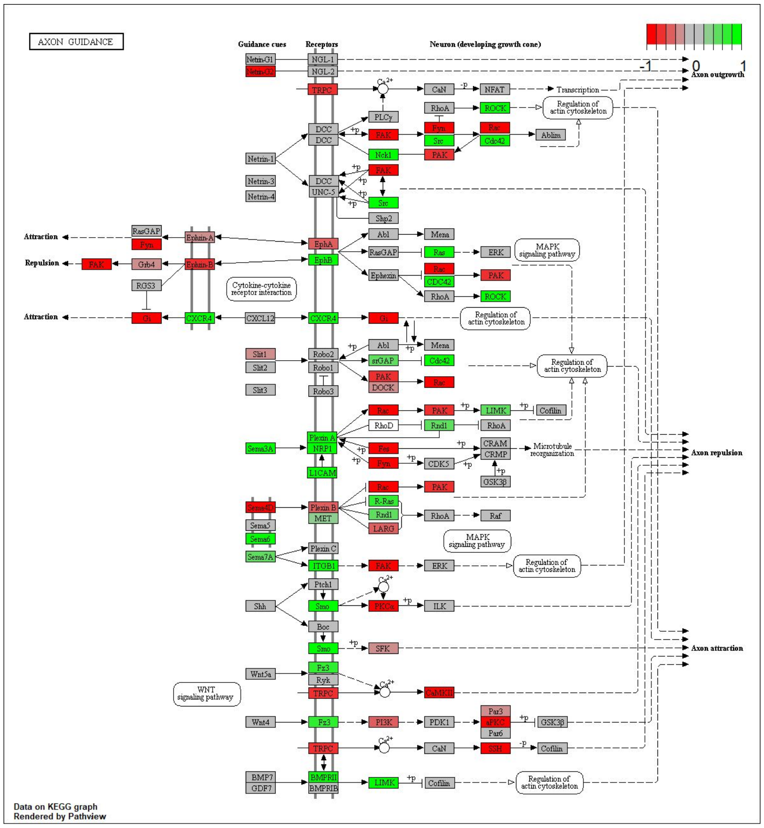

Figure 3

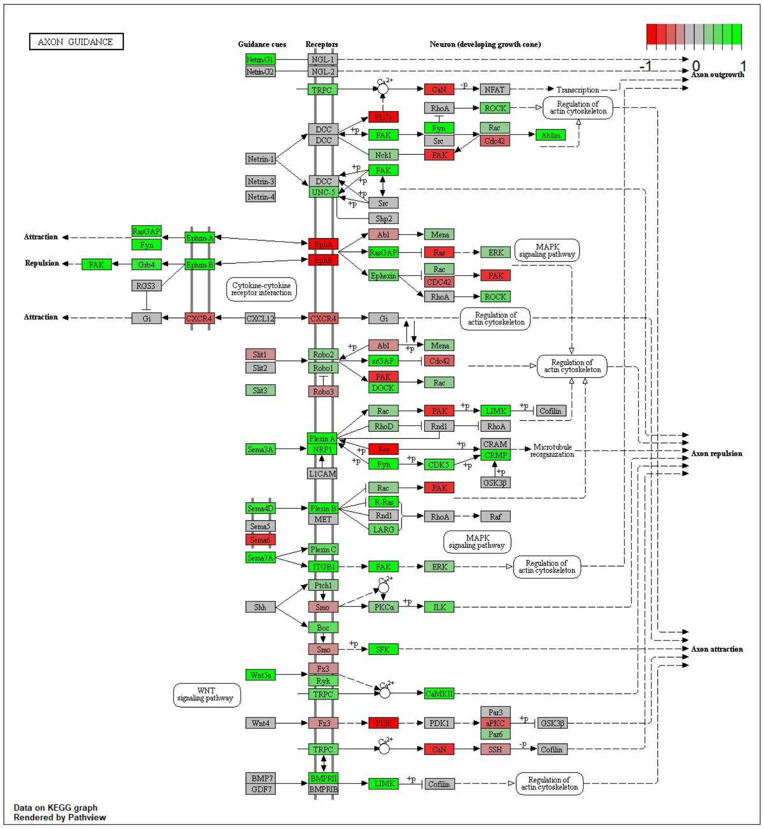

Figure 4

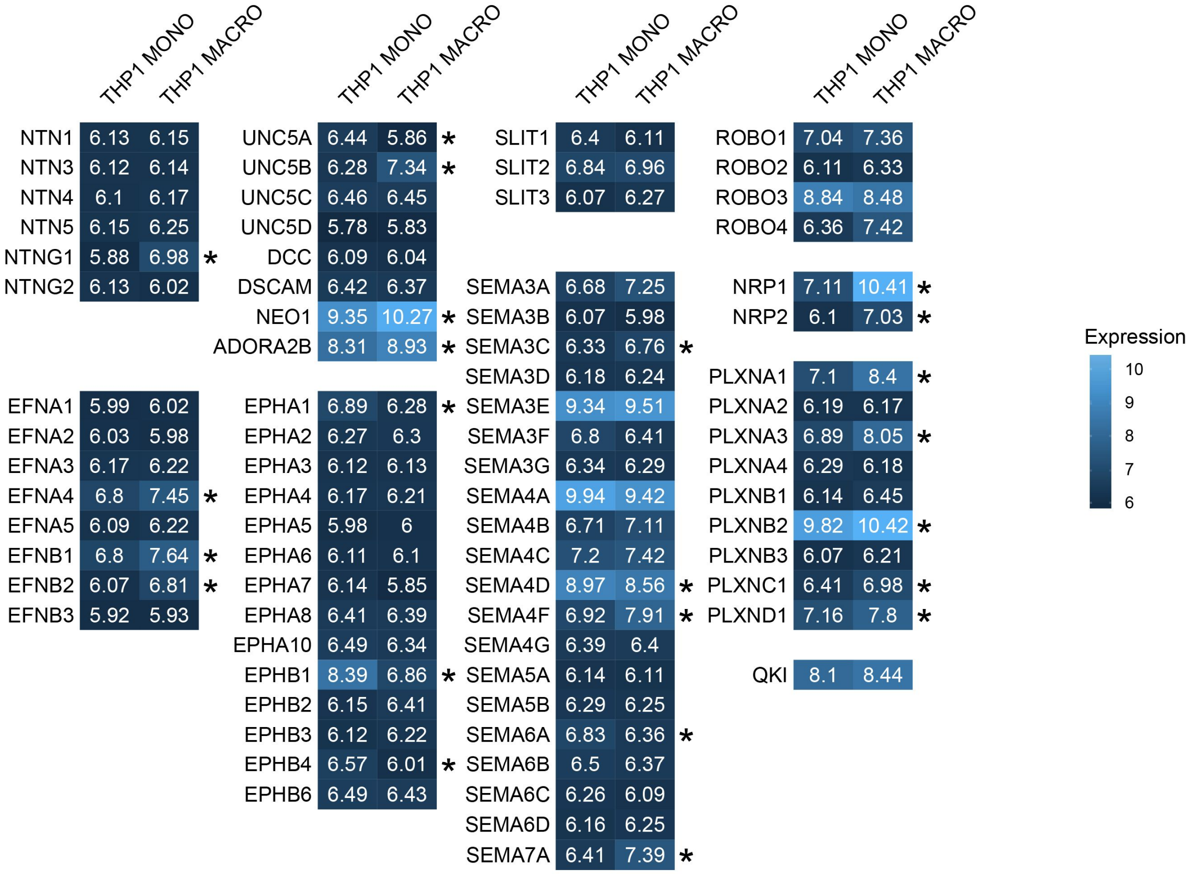
THP1 MONO
THP1 MACRO
NTN1 6.13 6.15
NTN3 6.12 6.14
NTN4 6.1 6.17
NTN5 6.15 6.25
NTNG1 5.88 6.98 *
NTNG2 6.13 6.02
EFNA1 5.99 6.02
EFNA2 6.03 5.98
EFNA3 6.17 6.22
EFNA4 6.8 7.45 *
EFNA5 6.09 6.22
EFNB1 6.8 7.64 *
EFNB2 6.07 6.81 *
EFNB3 5.92 5.93
UNC5A 6.44 5.86 *
UNC5B 6.28 7.34 *
UNC5C 6.46 6.45
UNC5D 5.78 5.83
DCC 6.09 6.04
DSCAM 6.42 6.37
NEO1 9.35 10.27 *
ADORA2B 8.31 8.93 *
EPHA1 6.89 6.28 *
EPHA2 6.27 6.3
EPHA3 6.12 6.13
EPHA4 6.17 6.21
EPHA5 5.98 6
EPHA6 6.11 6.1
EPHA7 6.14 5.85
EPHA8 6.41 6.39
EPHA10 6.49 6.34
EPHB1 8.39 6.86 *
EPHB2 6.15 6.41
EPHB3 6.12 6.22
EPHB4 6.57 6.01 *
EPHB6 6.49 6.43
SLIT1 6.4 6.11
SLIT2 6.84 6.96
SLIT3 6.07 6.27
SEMA3A 6.68 7.25
SEMA3B 6.07 5.98
SEMA3C 6.33 6.76 *
SEMA3D 6.18 6.24
SEMA3E 9.34 9.51
SEMA3F 6.8 6.41
SEMA3G 6.34 6.29
SEMA4A 9.94 9.42
SEMA4B 6.71 7.11
SEMA4C 7.2 7.42
SEMA4D 8.97 8.56 *
SEMA4F 6.92 7.91 *
SEMA4G 6.39 6.4
SEMA5A 6.14 6.11
SEMA5B 6.29 6.25
SEMA6A 6.83 6.36 *
SEMA6B 6.5 6.37
SEMA6C 6.26 6.09
SEMA6D 6.16 6.25
SEMA7A 6.41 7.39 *
ROBO1 7.04 7.36
ROBO2 6.11 6.33
ROBO3 8.84 8.48
ROBO4 6.36 7.42
NRP1 7.11 10.41 *
NRP2 6.1 7.03 *
PLXNA1 7.1 8.4 *
PLXNA2 6.19 6.17
PLXNA3 6.89 8.05 *
PLXNA4 6.29 6.18
PLXNB1 6.14 6.45
PLXNB2 9.82 10.42 *
PLXNB3 6.07 6.21
PLXNC1 6.41 6.98 *
PLXND1 7.16 7.8 *
QKI 8.1 8.44
Expression
10
9
8
7
6

Figure 5

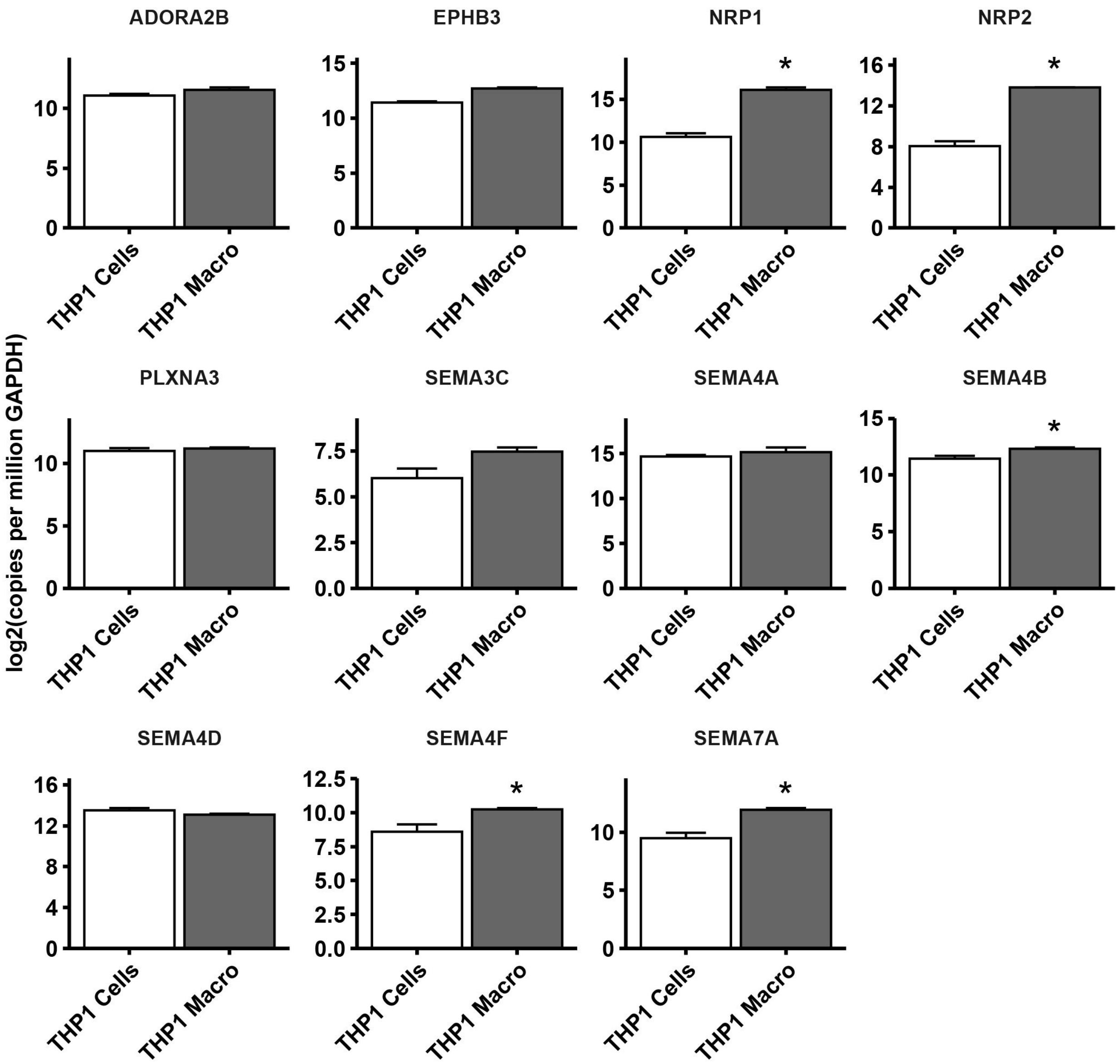
ADORA2B
EPHB3
NRP1
NRP2
PLXNA3
SEMA3C
SEMA4A
SEMA4B
SEMA4D
SEMA4F
SEMA7A
log2(copies per million GAPDH)
THP1 Cells
THP1 Macro
*

Figure 6
