# Supplementary material for: Comprehensive analysis of neuronal guidance cue expression regulation during monocyte-to-macrophage differentiation reveals post-transcriptional regulation of semaphorin7A by the RNA-binding protein quaking
Source: Innate Immun. 2020 Nov 26;27(2):118–32. doi: 10.1177/1753425920966645 (PMC7882812; doi:10.1177/1753425920966645)
Supplement: sj-pdf-2-ini-10.1177_1753425920966645 - Supplemental material for Comprehensive analysis of neuronal guidance cue expression regulation during monocyte-to-macrophage differentiation reveals post-transcriptional regulation of semaphorin7A by the RNA-binding protein quaking [file sj-pdf-2-ini-10.1177_1753425920966645.pdf]

Supplementary Table 1. Summary of the data accessions used in relation to the experimental materials and the profiling methods.

No.	Material	Description	Type	Accession	Differentiation Method	Profiling Method
1	Human monocyte	Patients with atherosclerosis and healthy controls	online	GSE9820	20 h Plastic Culture	Microarray (mRNA)
2	THP-1 cell line	THP-1 cells and THP-1 macrophage	in house	GSE74887	PMA (100 n) for 8 d	Microarray (mRNA)
3	Human monocyte	QKI haploinsufficiency patient and her sibling	in house	GSE74978	GM-CSF (5 ng/ml) for 7 d	RNAseq (mRNA)
4	Human monocyte	Healthy controls	online	GSE52986	NA	Microarray (miRNA)
5	K562 cell line	Isogenic duplicates of QKI eCLIP-seq in K562 cells	online	ENCSR366YOG	NA	eCLIP-seq
